# The effectiveness of intervention measures on MERS-CoV transmission by using the contact networks reconstructed from link prediction data

**DOI:** 10.3389/fpubh.2024.1386495

**Published:** 2024-05-17

**Authors:** Eunmi Kim, Yunhwan Kim, Hyeonseong Jin, Yeonju Lee, Hyosun Lee, Sunmi Lee

**Affiliations:** ^1^Institute of Mathematical Sciences, Ewha Womans University, Seoul, Republic of Korea; ^2^College of General Education, Kookmin University, Seoul, Republic of Korea; ^3^Department of Mathematics, Jeju National University, Jeju, Republic of Korea; ^4^Division of Applied Mathematical Sciences, Korea University—Sejong, Sejong, Republic of Korea; ^5^Applied Mathematics, Kyung Hee University, Yongin, Republic of Korea

**Keywords:** MERS-CoV, link prediction, network-based models, interventions, graph autoencoder (GAE)

## Abstract

**Introduction:**

Mitigating the spread of infectious diseases is of paramount concern for societal safety, necessitating the development of effective intervention measures. Epidemic simulation is widely used to evaluate the efficacy of such measures, but realistic simulation environments are crucial for meaningful insights. Despite the common use of contact-tracing data to construct realistic networks, they have inherent limitations. This study explores reconstructing simulation networks using link prediction methods as an alternative approach.

**Methods:**

The primary objective of this study is to assess the effectiveness of intervention measures on the reconstructed network, focusing on the 2015 MERS-CoV outbreak in South Korea. Contact-tracing data were acquired, and simulation networks were reconstructed using the graph autoencoder (GAE)-based link prediction method. A scale-free (SF) network was employed for comparison purposes. Epidemic simulations were conducted to evaluate three intervention strategies: Mass Quarantine (MQ), Isolation, and Isolation combined with Acquaintance Quarantine (AQ + Isolation).

**Results:**

Simulation results showed that AQ + Isolation was the most effective intervention on the GAE network, resulting in consistent epidemic curves due to high clustering coefficients. Conversely, MQ and AQ + Isolation were highly effective on the SF network, attributed to its low clustering coefficient and intervention sensitivity. Isolation alone exhibited reduced effectiveness. These findings emphasize the significant impact of network structure on intervention outcomes and suggest a potential overestimation of effectiveness in SF networks. Additionally, they highlight the complementary use of link prediction methods.

**Discussion:**

This innovative methodology provides inspiration for enhancing simulation environments in future endeavors. It also offers valuable insights for informing public health decision-making processes, emphasizing the importance of realistic simulation environments and the potential of link prediction methods.

## 1 Introduction

The spread of infectious diseases is an issue of paramount societal significance. As evidenced by the profound impact of the recent COVID-19 pandemic, the failure to effectively prevent or mitigate disease transmission can result in substantial social and economic costs. Therefore, it is imperative for the safety of society to uncover the fundamental mechanisms underlying infectious diseases and devise preventive measures accordingly. Collaborative efforts across various academic disciplines have been directed toward this goal. Insights from fields such as medicine, pharmacy, biology, engineering and sciences, and even social sciences play a crucial role in enhancing our understanding of disease transmission dynamics. Of particular importance is understanding how individuals engage in physical interactions, as disease transmission is intricately linked to the types and patterns of human contacts. Consequently, there have been concerted efforts to quantitatively describe and analyze human contacts within the context of disease transmission. One of the representatives has been network models (1, 2). Infected cases and their contacts were represented as nodes and links, respectively, on a network, and the network was analyzed to obtain insights into the transmission process (3, 4). In addition, a network can offer an environment for epidemic simulations, allowing for the acquisition of new knowledge not possible with compartmental model simulations (5). Contact-tracing data can be conducive to generating environments for epidemic simulations. The transmission dynamics of a network highly depends on the network structure, and networks generated based on real human behaviors can provide opportunities to build more realistic models of transmission dynamics in the literature (6–9) than the theoretical models of networks such as random (10, 11), small-world (12, 13), and scale-free (SF) (14, 15) networks.

However, using empirical contact-tracing data for epidemic simulation has limitations (16, 17). One theoretical limitation arises from the fact that individuals have numerous social relationships, but only a portion of these relationships result in actual contacts in practice. In other words, the contacts that individuals make represent only a subset of the many possibilities within their social connections. When conducting simulations, we essentially explore artificial scenarios where social relationships could have played out differently from the real world. Therefore, the network environments used in simulations should reflect the range of possibilities within these relationships, rather than replicating a single, actual realization. Conducting simulations on a contact-tracing network may involve exploring artificial scenarios based on a specific realization, which can pose logical challenges. Another limitation is of an empirical nature. Real-world data, including contact-tracing data, are inherently affected by noise. Contact information can be collected through various means, such as self-reports, cell phone location tracking, or third-party observations. However, noise originating from human errors or technical inaccuracies can result in missing nodes or links within contact-tracing networks. This missing or inaccurate data can affect the reliability and accuracy of simulations that rely on such data. Therefore, using empirical contact-tracing data for epidemic simulations has limitations related to both theoretical considerations, where simulations explore artificial scenarios based on partial realizations, and empirical issues, including noise and missing data inherent in real-world contact-tracing information. Researchers and modelers need to be aware of these limitations and consider them when using such data for epidemiological simulations.

The aforementioned limitations can be complemented by network reconstruction (18). Network reconstruction entails generating a network from another network that has missing or spurious links in its observed status (19). It can be considered as correcting errors in network data because the observed network topology is compared with the theoretical models of network evolution (20). Among many, link prediction (LP) is a promising network reconstruction technique (21–23). LP entails estimating the probability of connecting two nodes that are currently not connected based on the linkage patterns and node features. Several techniques, including matrix factorization, the stochastic block model (24), DeepWalk (25), node2vec (26), and LINE (27), have been used for LP. Recently, advanced techniques in graph neural networks (GNNs) have been actively employed for LP, and the prediction accuracy has been significantly improved in various domains. Since the notion of GNN was initially devised (28), various learning models in the graph domain have been developed (29–31). Convolutional neural networks in the computer vision domain have been redefined for graph data and developed in parallel as convolutional GNNs (ConvGNNs) (29, 30). Recent studies have increased the capabilities and expressive power of ConvGNNs in various practical applications, such as antibacterial discovery (31), fake news detection (32), traffic prediction (33), and recommendation systems (34).

Based on the above considerations, in this study, we reconstruct networks from real-world contact-tracing data and perform epidemic simulations on them. In particular, we examine how the network structure impacts the transmission dynamics and the effectiveness of intervention strategies. As a case study, we consider the Middle East Respiratory Syndrome Coronavirus (MERS-CoV) transmission in South Korea, 2015. Most existing studies on MERS-CoV focused on its epidemic characteristics and demonstrated its super-spreading events (35–37). Some other studies analyzed the contact-tracing data, but their networks were confined only to confirmed cases (38, 39), single hospitals (40), or regions without large-scale outbreaks (41). Thus, their contact networks were of limited use for epidemic simulations. Another study extracted the parameter of an SF network from MERS-CoV contact-tracing data and generated a simulation environment with it, but the contact network itself was not used for simulation (42). In this study, we construct simulation environments using reconstructed networks and examine the dynamics of the epidemic within these environments. Simulation involves utilizing a network generated through graph autoencoder-based link prediction (GAE network), and a scale-free (SF) network is employed for comparative analysis. Then, we conduct epidemic simulations to assess three intervention strategies: Mass Quarantine (MQ), Isolation, and Isolation combined with Acquaintance Quarantine (AQ + Isolation).

The remainder of this article is organized as follows. Section 2 introduces the empirical contact network of the 2015 MERS-CoV transmission in South Korea and demonstrates the network reconstruction for simulation environments. Section 3 presents the simulation procedure, and Section 4 describes the simulation results. The implications, and limitations of this study are discussed in Section 5. Finally, the conclusions are given in Section 6.

## 2 Materials and methods

### 2.1 Reconstructing the empirical contact network by link prediction

#### 2.1.1 Empirical contact network

**Figure 1:**
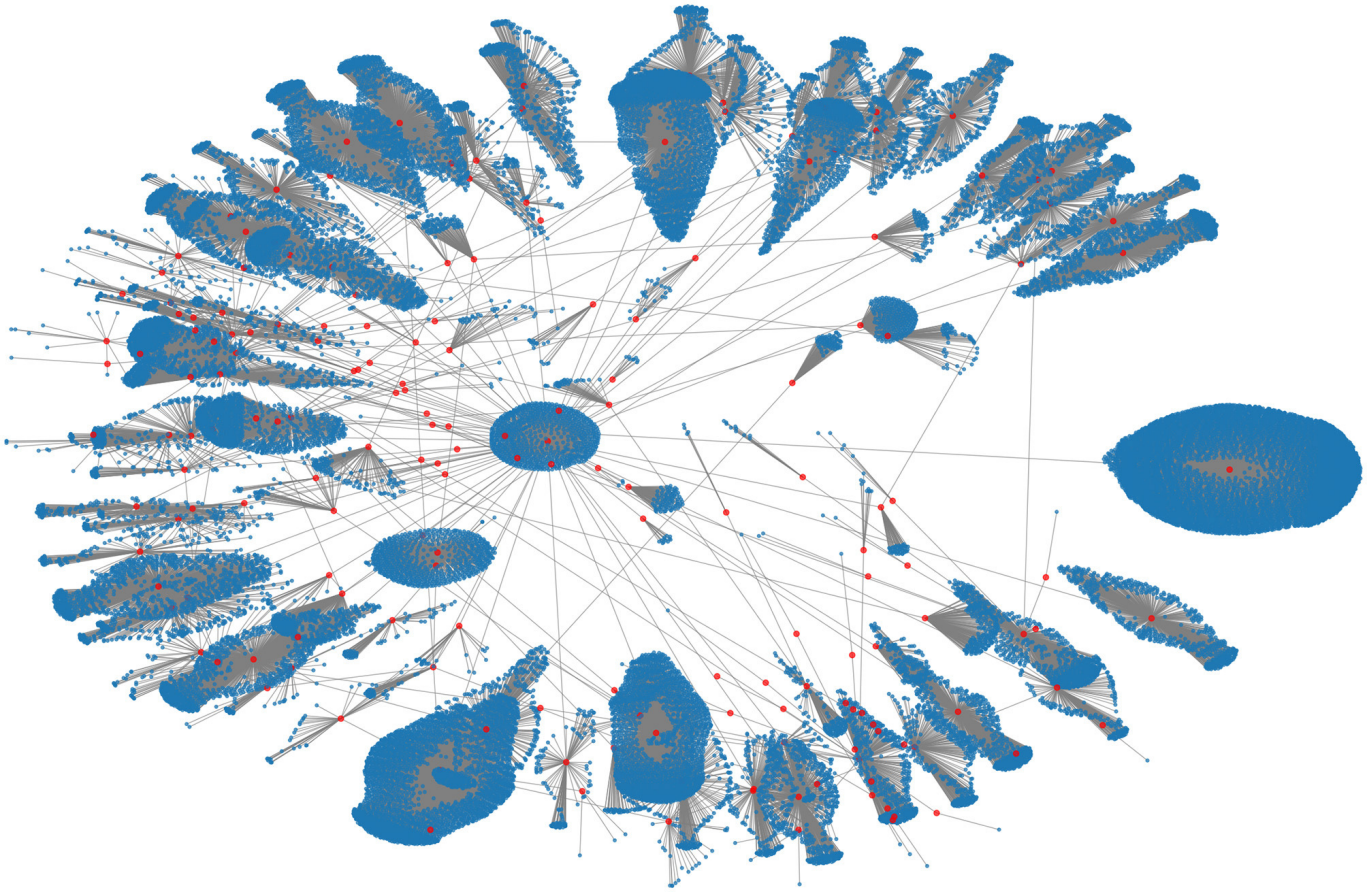
The empirical contact network of the 2015 MERS-CoV transmission in South Korea. The red and blue nodes denote 186 confirmed cases and their contact nodes (a total of 33,093), respectively.

The data for the 2015 MERS-CoV outbreak was obtained from the websites of the Korea Centers for Disease Control and Prevention (KCDC) and the Ministry of Health and Welfare of South Korea (43). These data included information on confirmed cases and their contacts. A network was created from this information, which consisted of 33,093 nodes and 33,090 links, with 186 confirmed cases represented as red nodes and the individuals who had close or casual contact with them represented as blue nodes. Figure 1 shows this contact network.

#### 2.1.2 Link prediction using graph autoencoder

**Figure 2:**
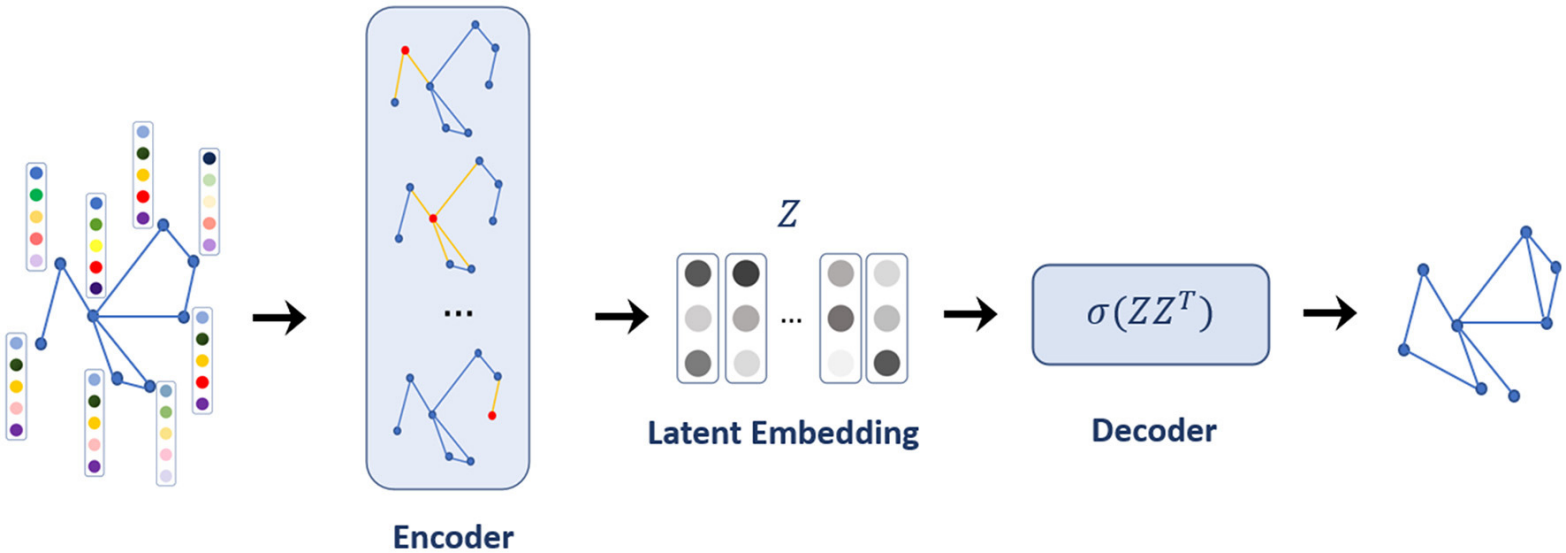
The model architecture of a graph autoencoder.

The generated contact network lacks some links between nodes due to some missing information in the data. Thus, the contact network was reconstructed by LP using a graph autoencoder. The graph autoencoder is a neural network model for learning interpretable latent representations of graph-structured data based on an autoencoder (44). In the graph autoencoder framework for LP, the encoder employs a graph convolutional network (GCN) incorporating node features for the latent embedding of each node. Then, the decoder computes the distance between two nodes in the given node embeddings, from which the occurrence of an edge between the two nodes is predicted (see Figure 2 for the model architecture).

Formally, for a graph *G* = (*V, E*) defined by a set of node *V* and a set of edges *E* between nodes, the encoder maps nodes *v*∈*V* with node features $x_{v}\in {\mathbb{R}}^{n}$ to latent embedding vectors $z_{v}\in {\mathbb{R}}^{d}$ with Equation (1):


(1) $$\begin{array}{l} \begin{array}{cc} ENC:V\times {\mathbb{R}}^{n} & \to {\mathbb{R}}^{d}\\ \left(v,x_{v}\right)\; & \mapsto z_{v}. \end{array} \end{array}$$

Employing the decoder for a pair of node embeddings (*z*_*u*_, *z*_*v*_) will estimate a graph-structured similarity score *S*[*u, v*] between nodes *u* and *v*. The objective of the encoder and decoder is to minimize the reconstruction loss such that Equation (2),


(2) $$\begin{array}{l} DEC\left(ENC\left(u\right),ENC\left(v\right)\right)=DEC\left(z_{u},z_{v}\right)\approx S\left[u,v\right]. \end{array}$$

For LP, the similarity score between nodes can be considered as representing whether nodes are neighbors or not; this means that node embeddings *z*_*u*_ and *z*_*v*_ are close in the embedding space if they are linked. The links in the contact network stand for the contact from confirmed cases to other individuals, and individuals in the same cluster may have more contacts with each other than with those in other clusters (the visualization of the contact network in Figure 1 shows the cluster structure). Thus, the cluster, as well as infection status, was used as node features to predict links between nodes using a graph autoencoder. A label propagation algorithm (45) was used for cluster analysis on the contact network, and 61 clusters were detected.

Our GCN model for the encoder has two graph convolution layers with a 256-dim hidden layer and 128-dim latent embedding space. A simple inner product was used for the decoder, which could provide a score as the probability of internode link occurrence, and the sigmoid function was used as the activation function. The model was trained for 500 iterations using an Adam optimizer with a learning rate of 0.005. The reconstructed networks were obtained from the ensemble of 10 trained models. Next, two types of networks were generated. First, a pair of nodes whose similarity score was >0.995 was connected by links; we name this network GAE. Second, as an extended version, a pair of nodes whose similarity score was >0.95 (lower than that of GAE) was connected by links; we name this network GAE_ex as we extend GAE. A similarity score of 0.95 was selected to attain a 99.99% accuracy in recovering existing links. Reducing the similarity score further did not lead to a significant improvement in accuracy. A score of 0.995 was employed for 0.95 networks when they were deemed excessively large (almost twice larger in edges). Increasing the similarity score results in the prediction of additional links. When viewed as a generative model for situations where the original contact network is unavailable, it becomes essential to generate a network of an appropriate scale for practical use.

We validate the accuracy of our graph encoder model in generating results that closely match the actual contact network. It is worth noting that GAE_ex, generated by our graph encoder model, reconstructs the contact network with an accuracy of 99.99% (missing only three edges out of the existing 33,090 edges) and generates 211,778 new possible edges. Similarly, GAE reconstructs the contact network with an accuracy of 98.92% (missing 359 edges out of the existing 33,090 edges) and generates 111,536 new possible edges. Subsequently, both GAE_ex and GAE are finalized by adding missing edges, likely aiming to enhance the completeness of the reconstructed networks. Both GAE_ex and GAE demonstrate effectiveness in reconstructing the contact network, with GAE_ex achieving slightly higher accuracy in capturing existing edges, while GAE generates fewer new possible edges. Therefore, we have selected these two networks for our simulation network.

#### 2.1.3 Properties of reconstructed networks

**Figure 3:**
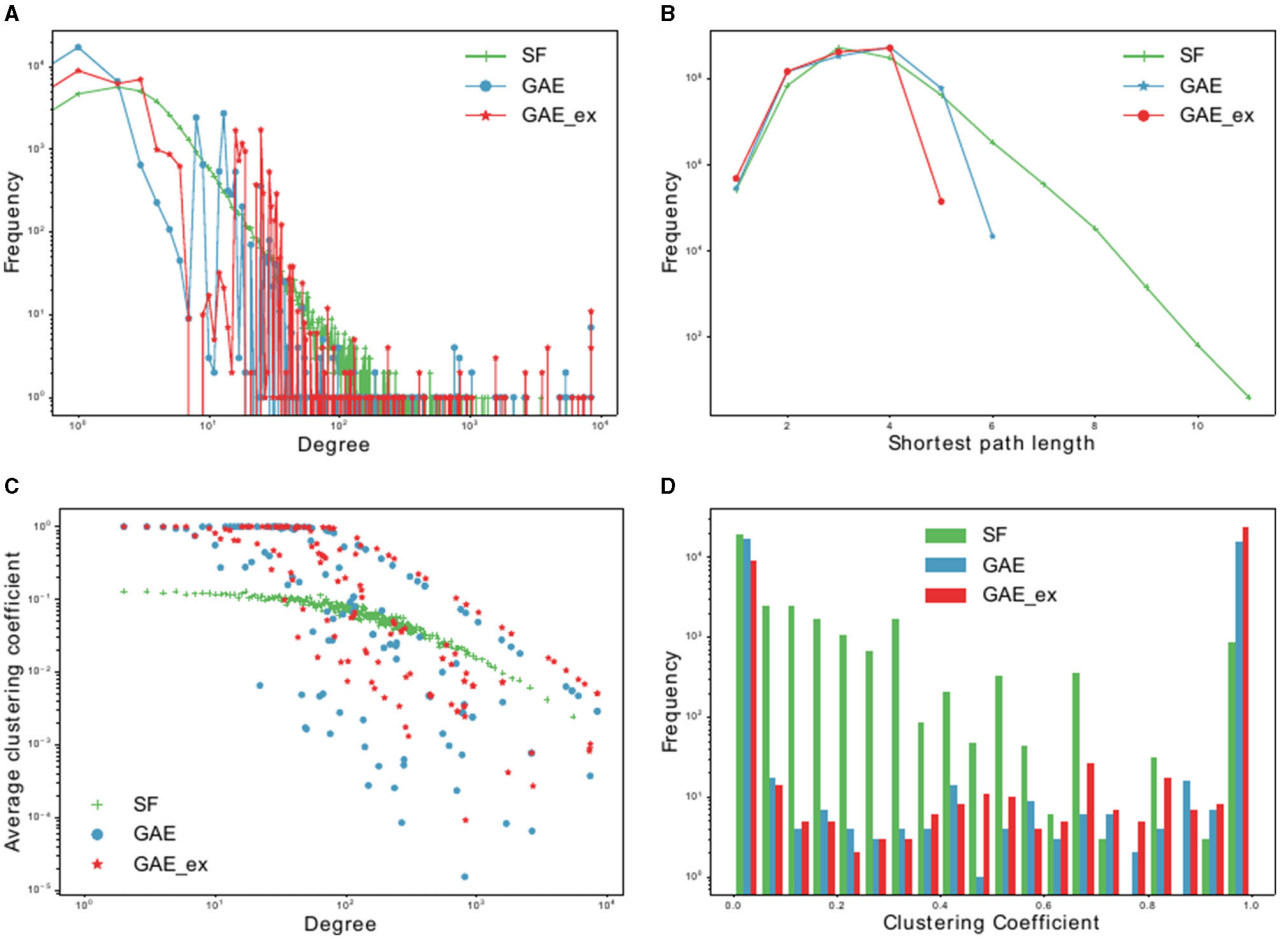
Basic characteristics of two reconstructed contact networks and scale-free (SF) network. **(A)** Degree distributions, **(B)** distribution of shortest path lengths, **(C)** clustering coefficient distributions per degree, and **(D)** clustering coefficient distributions.

**Figure 4:**
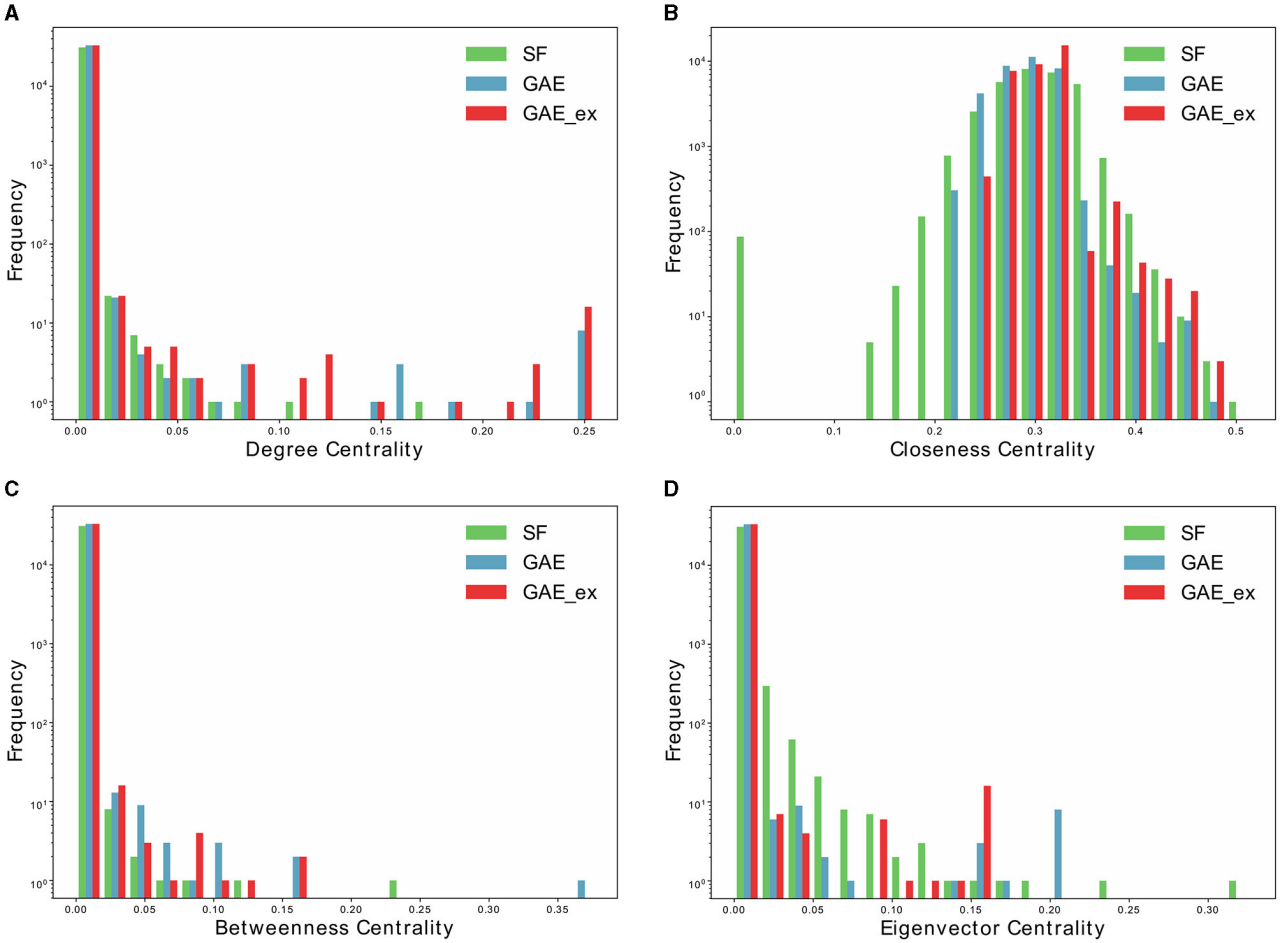
Distributions of centrality indexes of two reconstructed contact networks and scale-free (SF) network. **(A)** Degree centrality distribution, **(B)** closeness centrality distribution, **(C)** betweenness centrality distribution, and **(D)** Eigenvector centrality distribution.

In this section, we analyzed the characteristics of the networks reconstructed using the graph autoencoder before running the simulations. We opted for the scale-free network for comparative purposes. By conducting simulations on both types of networks, we aimed to illustrate the differences in disease spread within the reconstructed networks compared to the well-understood scale-free network. We chose the scale-free network because it has been more commonly employed in the literature than other models, such as the random-network model, making it more suitable for meaningful comparisons. First, we fitted the degree distribution of the GAE network to the power-law distribution, and found that the scale parameter was 2.19. We then used this parameter to generate a scale-free network using the configuration model (46). The main properties of the scale-free, GAE, and GAE_ex networks are outlined in Tables 1, 2 and Figures 3, 4.

**Table 1:** Basic characteristics of two reconstructed contact networks and scale-free (SF) network.

	**Nodes**	**Edges**	**Avg. degree**	**Connected components**	**Avg. shortest path length**	**Avg. clustering coefficient**
SF	31,091	132,372	8.5151	44	3.3481	0.1037
GAE	33,093	144,626	8.7406	1	3.4727	0.4825
GAE_ex	33,093	244,868	14.7988	1	3.3386	0.7275

**Table 2:** Comparison of centrality indexes among two reconstructed contact networks and scale-free (SF) network.

	**Degree centrality**		**Closeness centrality**		**Betweenness centrality**		**Eigenvector centrality**	
	**Avg**.	**Cent(%)**	**Avg**.	**Cent(%)**	**Avg**.	**Cent(%)**	**Avg**.	**Cent(%)**
SF	0.000274	17.6450	0.301515	42.8943	0.000075	4.91e-8	0.002145	46.0836
GAE	0.000264	25.4405	0.291004	37.7405	0.000075	6.87e-8	0.002006	29.8501
GAE_ex	0.000447	25.4282	0.301885	37.0357	0.000071	3.01e-8	0.002044	22.4648

We compare the properties of networks generated using a graph autoencoder (GAE), an extended version of the GAE (GAE_ex), and a scale-free (SF) model. The average degree of GAE_ex is found to be significantly greater than that of GAE and SF in Table 1. All three networks have similar degree distributions, with few nodes having much greater degrees than others as shown in Figure 3A. GAE and GAE_ex have higher average clustering coefficients than SF, indicating that the reconstructed networks have a highly clustered structure similar to those found in real-world social networks (47). Additionally, while SF had multiple disconnected components (a total of 44 connected components), GAE and GAE_ex were fully connected. The average shortest path length for all three networks is short (see Figure 3B and Table 1), making them small-world networks. However, SF is found to have a larger diameter than GAE and GAE_ex, indicating that some pairs of nodes in SF are connected by larger hops. The reconstructed networks, GAE and GAE_ex, exhibit greater variation in the average clustering coefficients per degree when compared to the SF network (as shown in Figures 3C, D). This suggests that the connections among the neighbors of nodes with similar degrees are more varied in GAE and GAE_ex than in SF. Additionally, even though the average degrees of GAE and SF are similar, their distributions of average clustering coefficients differ.

Furthermore, Table 2 and Figure 4 present average centrality and centralization index using four different centrality measures in the three networks. Average centrality reflects the characteristics of each node in the network, while the centralization index assesses the distribution of centrality. A higher centralization index suggests a more centralized network, while a lower index indicates a more evenly distributed centrality (see distributions of centrality in Figure 4). Specifically, the average degree centrality for GAE_ex is 0.00044, which is twice as high as that of the GAE and SF networks presented in Table 2. This indicates that, on average, nodes in the GAE_ex network have approximately twice as many connections compared to nodes in the GAE and SF networks. For the other three centrality measures, there are no significant differences between the SF and GAE networks. Closeness centrality, which measures how well-connected a node is to all other nodes, shows similar low values in both networks. Eigenvector centrality, indicating the level of influence of nodes within their respective networks, is similar for nodes in both networks, and it suggests a relatively low level of influence on average. Betweenness centrality, which assesses the role of nodes as intermediaries or bridges between others, also shows similar low values for nodes in both the SF and GAE networks, indicating a limited intermediary role on average. In summary, the analysis of these centrality measures suggests that while the average degree centrality differs significantly between GAE_ex and the other networks, the other three centrality measures (closeness, eigenvector, and betweenness centrality) do not reveal significant distinctions between the SF and GAE networks. This implies that, in terms of these specific centrality metrics, the networks share similarities in how nodes are connected and their roles within the network.

### 2.2 Simulation model

We employed agent-based simulations on the generated networks as reported in Kim et al. (42). The simulations were based on the SEIR model, where each agent (node) was assigned one of four epidemiological statuses: susceptible (*S*), exposed (*E*), infected (*I*), and recovered (*R*). The simulation assumed that when a susceptible agent comes into contact with an exposed or infected agent, it has a probability of becoming infected with a transmission rate of β with below Equation (3):


(3) $$\begin{array}{l} \beta =1-{\left(1-\beta_{0}\right)}^{n} \end{array}$$

In the simulations, an infection was generated when a susceptible individual came into contact with an exposed or infected individual, with a transmission probability determined by the transmission rate constant, β given above. The transmission rate was modeled as a function of the number of neighbors in the network and a baseline transmission constant, β_0_. The simulations also accounted for the incubation and infectious periods, which were modeled as gamma probability density functions with means of 1/κ and 1/γ days and standard deviations of σ_κ_ and σ_γ_ days, respectively. The parameters used in the model were estimated from confirmed cases data from the Korean Centers for Disease Control and Prevention (KCDC) (43) and are listed in Table 3.

**Table 3:** Model parameters and their values.

**Parameter**	**Description**	**Value**	**References**
*S*	Susceptible individual	–	–
*E*	Exposed individual	–	–
*I*	Infected individual	–	–
*R*	Recovered individual	–	–
*N*	Total population size	31,091	–
*T*	Total simulation time	100 day	–
β_0_	Background transmission constant	0.002	Estimated
*n*	A number of infected neighbors	–	–
β	Transmission rate	$\beta =1-{\left(1-\beta_{0}\right)}^{n}$	(42)
1/κ	Mean incubation period	8.7	(42)
σ_κ_	Standard deviation of the incubation period	16	(42)
1/γ	Mean infectious period	21	(42)
σ_γ_	Standard deviation of the infectious period	76	(42)

At the initialization phase of each simulation run, all agents, except an index case (the first infected agent), are set to be S status, and the predetermined index case is set to be I status. The index case is selected among the agents (nodes) with a sufficient number of links; an outbreak does not occur when the index agent is too far from the hub (when a node with a small degree is selected as the index case). Based on the preliminary experiments, the threshold of degree for selecting an index case was set to 100. The number of agents with more than 100° was 232 (out of 31,901) in SF, 80 (out of 33,093) in GAE, and 109 (out of 33,093) in GAE_ex; the index case for each simulation run is randomly chosen among them.

The intervention strategies in our research were developed based on an extensive review of existing literature on mathematical models of disease transmission (48–51). Previous studies have incorporated a variety of intervention measures into their models, with a specific focus on social distancing as a key strategy. Social distancing aims to reduce the chances of contact between individuals and has been a major topic of research in disease modeling (52, 53). In our study, we initially emphasized the “Mass Quarantine” strategy, which involves quarantining a certain percentage of the population (53). This strategy serves as an abstraction of real-world measures that restrict social activities, such as store closures and changes in public transportation operations. We selected a parameter of 10% for this strategy, guided by prior research (54).

We also introduced an “Isolation” strategy, which isolates individuals who have tested positive for the infection. This approach is conceptually similar to targeted social distancing, as it specifically targets infected individuals (52). While it may seem unrealistic to isolate all infected individuals, it was observed during the 2015 MERS-CoV outbreak and the early stages of the COVID-19 pandemic in South Korea, where infected individuals voluntarily isolated at home or were hospitalized under government guidance. Implicitly, the first two strategies were included for the purpose of comparison to assess the effectiveness of our third strategy: “Isolation and Acquaintance Quarantine (AQ + Isolation).” This approach involves quarantining individuals who have had close and effective contact with infectious individuals. We selected a parameter of 50% for this strategy, informed by previous research findings.

These intervention strategies are central to our study, allowing us to evaluate and compare their effectiveness in controlling disease spread. Specifically, the following three intervention strategies were investigated in terms of their effectiveness. Mass Quarantine (MQ): quarantining 10% of randomly chosen agents from S and E statuses; Isolation: isolating all agents from I status; and Isolation combined with Acquaintance Quarantine (AQ + Isolation): isolating all confirmed cases (individuals from I status) and quarantining 50% of randomly chosen agents from all agents who had effective contact with infected individuals. The intervention began on day 10 in each simulation run. Owing to the stochastic nature of agent-based models, all simulations were run 1,000 times, and their epidemic outputs were obtained.

## 3 Results

### 3.1 The impacts of intervention strategies and different network structure

**Figure 5:**
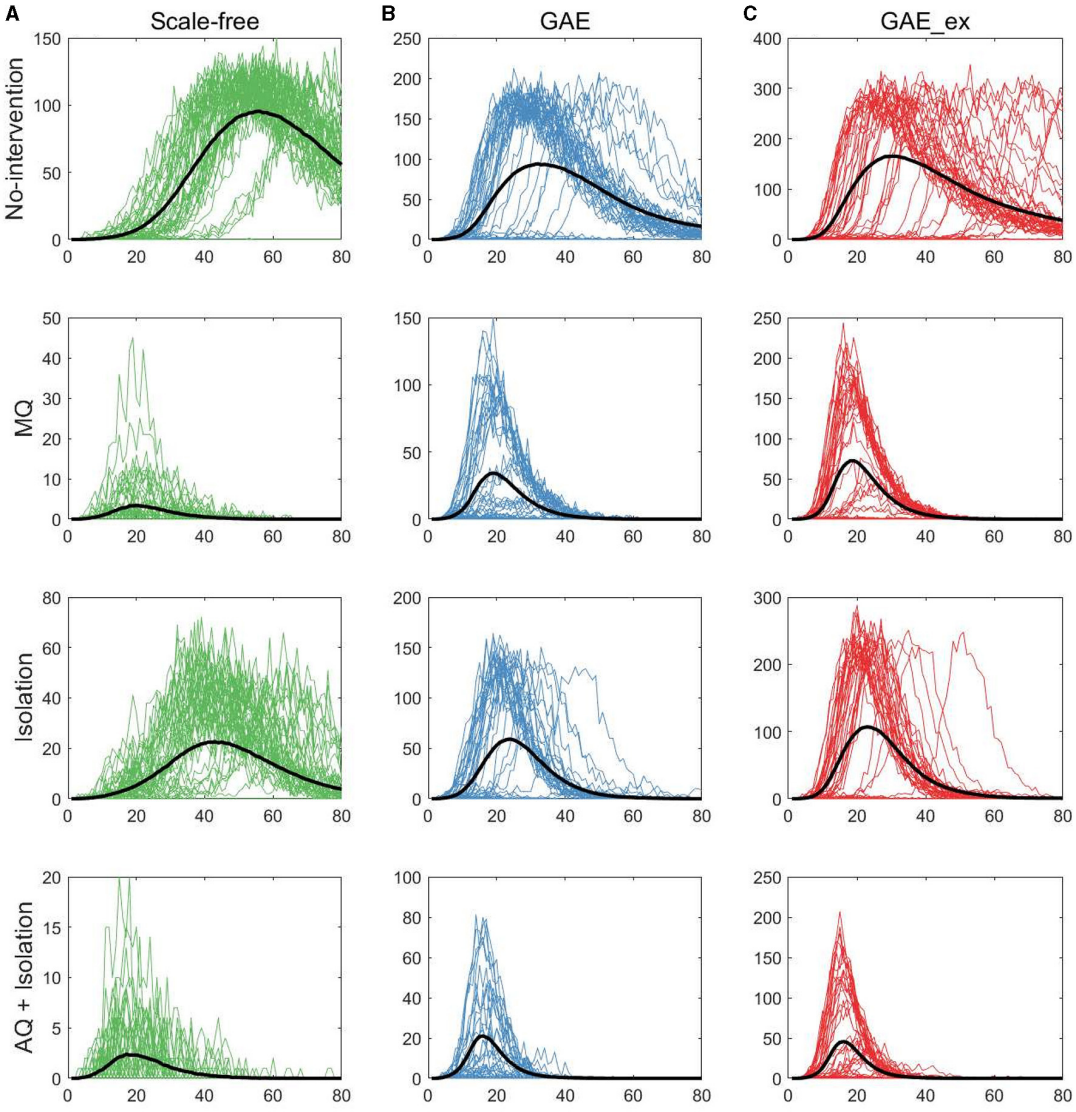
Daily incidences on three contact networks: **(A)** scale-free (SF), **(B)** GAE, and **(C)** GAE_ex [for each plot, 50 realizations of incidence are displayed with the mean (black curve). Each row shows the results for four distinct intervention strategies; top rows with a much larger peak size are the results with no intervention].

**Figure 6:**
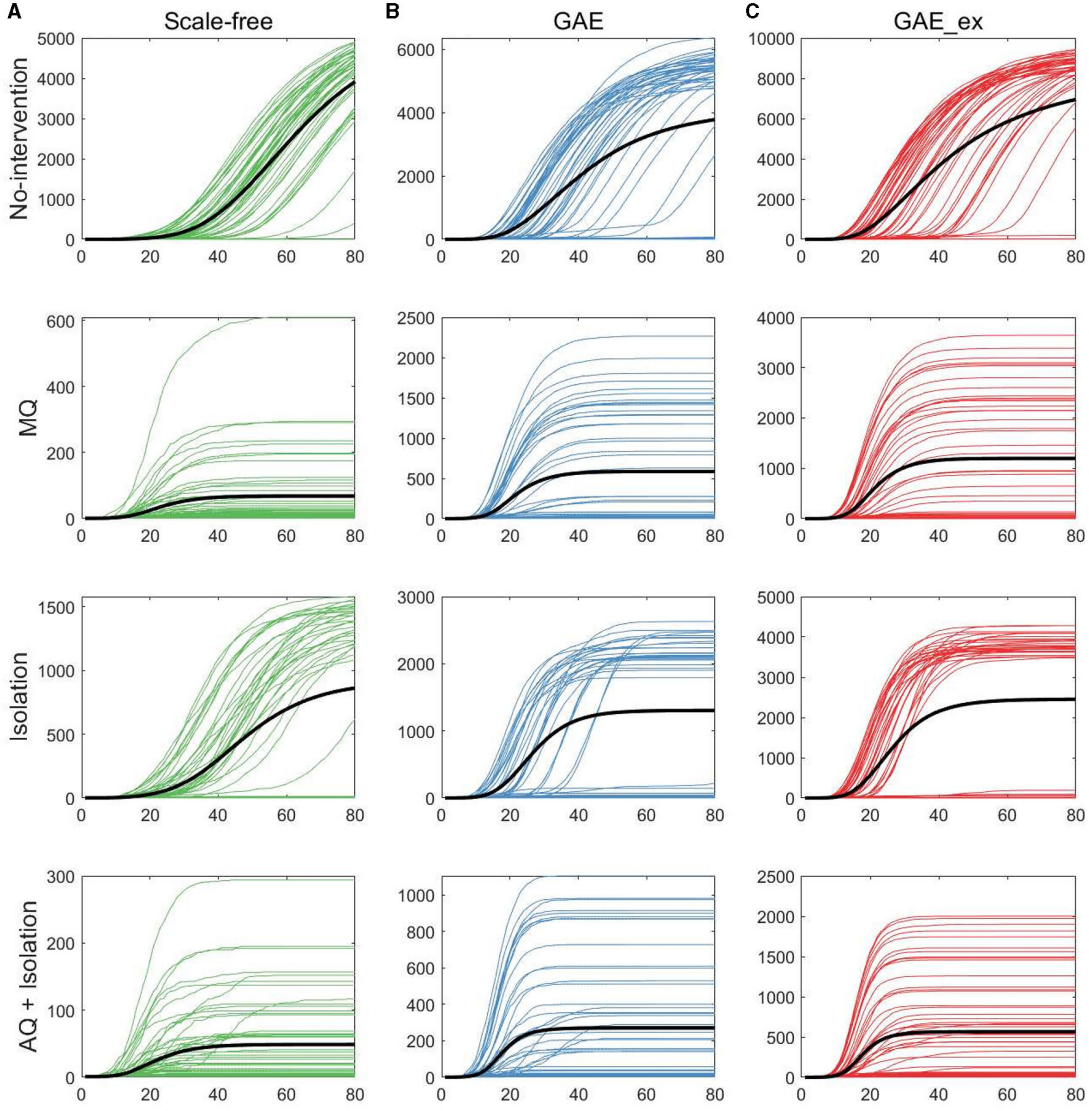
Cumulative incidences on three contact networks: **(A)** scale-free (SF), **(B)** GAE, and **(C)** GAE_ex [for each plot, 50 realizations of incidence are displayed with the mean (black curve). Each row shows the results for four distinct intervention strategies; top rows with a much larger peak size are the results with no intervention].

In this section, we investigate the impact of different network structures and intervention strategies on epidemic outputs. First, we present epidemic curves of MERS-CoV transmission dynamics: daily incidence in Figure 5 and cumulative incidence in Figure 6. The columns of the figures show the dynamics of three network structures: (a) SF (green), (b) GAE (blue), and (c) GAE_ex (red). Meanwhile, the rows show the dynamics for four intervention scenarios: No intervention, MQ, Isolation, and AQ + Isolation. Owing to the stochastic nature of our agent-based epidemic model, each result displays 50 realizations with the mean (black curve).

The first row of Figure 5 shows the impact of different network structures in the absence of interventions; it indicates that the outbreak gets worse in the order of SF, GAE, and GAE_ex, the peak size gets larger in that order (around 100, 200, and 300, respectively), and the peak time occurs earlier in GAE and GAE_ex (around day 30) than in SF (around day 60), attributable to the shortest path length (see Figure 3B). In addition, larger variances in the epidemic curve are observed in GAE (blue) and GAE_ex (red) than in SF (green), attributable to the larger variance in degree distributions and their clustering coefficients (Figures 3A, C).

**Figure 7:**
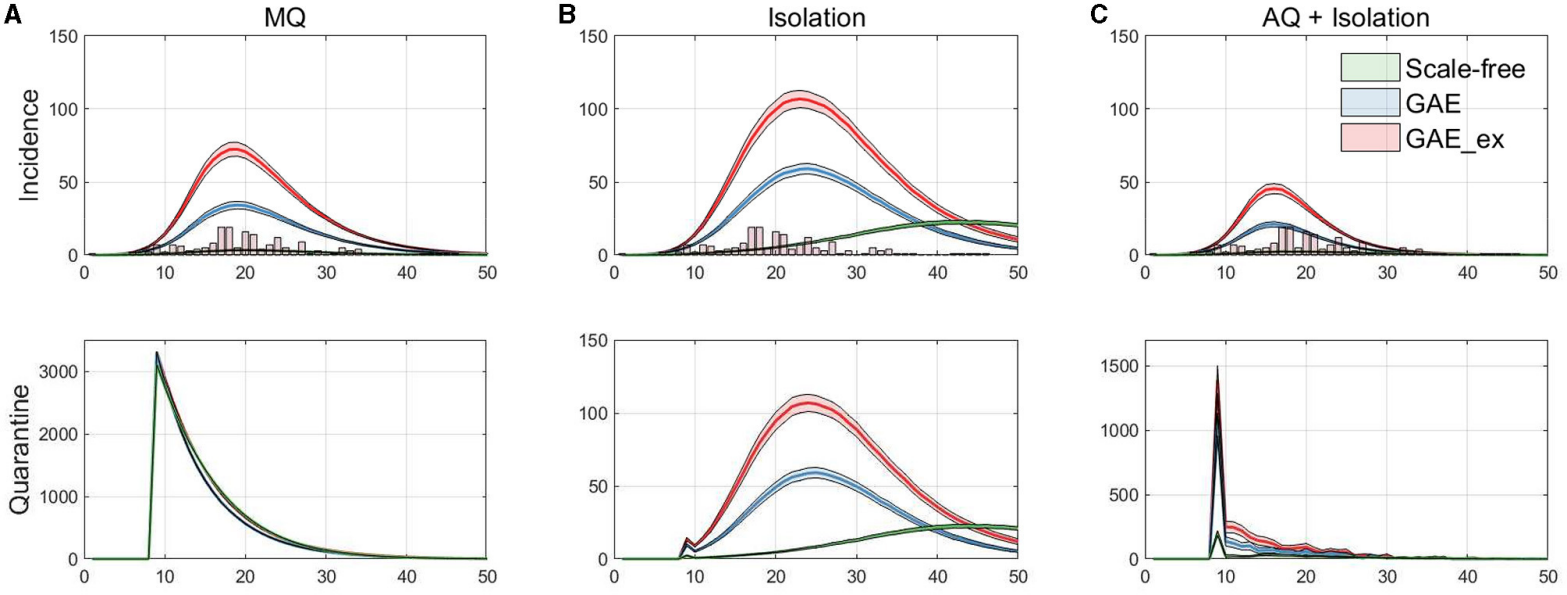
Daily incidences displayed with MERS-CoV data in vertical bar (top row), and the corresponding daily quarantined individuals (bottom row). **(A)** Mass Quarantine (MQ), **(B)** Isolation, and **(C)** Acquaintance Quarantine (AQ) + Isolation.

Next, the impact of the three intervention strategies is shown from the second to the last rows in Figure 5, which indicates that the daily incidence gets larger in the order of SF, GAE, and GAE_ex for all intervention strategies. In addition, from the second row, MQ reduced incidence in SF much more dramatically than in GAE and GAE_ex, attributable to the average and variance of clustering coefficients being much smaller in SF than in GAE and GAE_ex [recall that the average clustering coefficient is 0.1037, 0.4825, and 0.7275 for SF, GAE, and GAE_ex, respectively (Table 1), and see variances in Figure 3C]. Further, these results suggest that the most effective intervention is AQ + Isolation in all three network structures (see the bottom panels), with the earliest peak time and smallest peak size in all three networks. Besides, for AQ + Isolation, the most dramatic reduction of incidence was observed in SF than in GAE and GAE_ex. Isolation is the least effective in all network structures because only infected individuals are isolated without any contact-tracing and quarantine. The effectiveness of the three interventions is further described in Figure 7.

The results in Figure 6 show that, as in daily incidence in Figure 5, the variances of the cumulative incidences were larger in GAE and GAE_ex than in SF regardless of the intervention strategy; the 50 realization curves are less centered around the black mean curve in GAE and GAE_ex than in SF. Notably, for Isolation, the 50 realization curves generated bimodal results in GAE and GAE_ex: the black mean curves are placed between high and low cumulative incidences. These detailed epidemic outputs are further explored in the next subsection.

Finally, the incidence dynamics are compared with actual MERS-CoV incidence data and the number of quarantined individuals in the simulations. Figure 7 shows the averaged dynamics (mean of 1,000 realizations) of incidences on the three networks with actual MERS-CoV incidence data (histogram) at the top panels and the number of quarantined individuals at the bottom panels. Each column in the figure shows the results for the MQ, Isolation, and AQ + Isolation intervention strategies. The results in the figure suggest that MQ requires the maximum level of quarantine at the beginning for all three networks. In addition, although a similar number of individuals are quarantined under all networks, MQ is the most effective strategy for SF than GAE and GAE_ex (see green curves). Obviously, AQ + Isolation is the most effective intervention strategy for incidence reduction in all three networks (see the epidemic curves in the top panel). This strategy combines contact-tracing with quarantine; thus, much fewer people than in the MQ intervention are quarantined but much fewer people are infected.

### 3.2 The impacts of three network on various epidemic outputs

**Figure 8:**
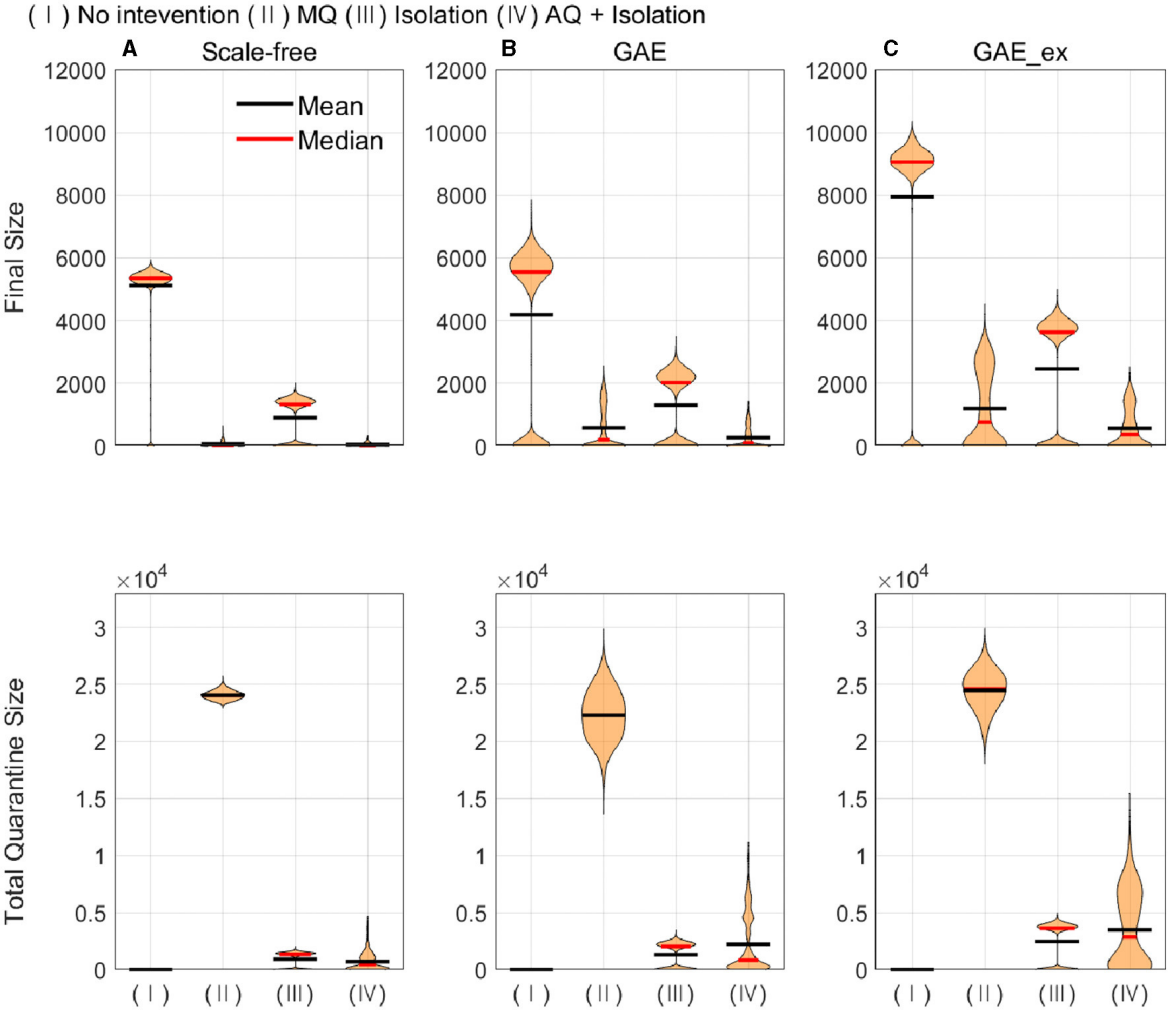
Cumulative infected (top row) and quarantined individuals (bottom row) under different intervention scenarios and on different networks (for each intervention, the result of 1,000 runs is shown). **(A)** SF, **(B)** GAE, and **(C)** GAE_ex.

**Figure 9:**
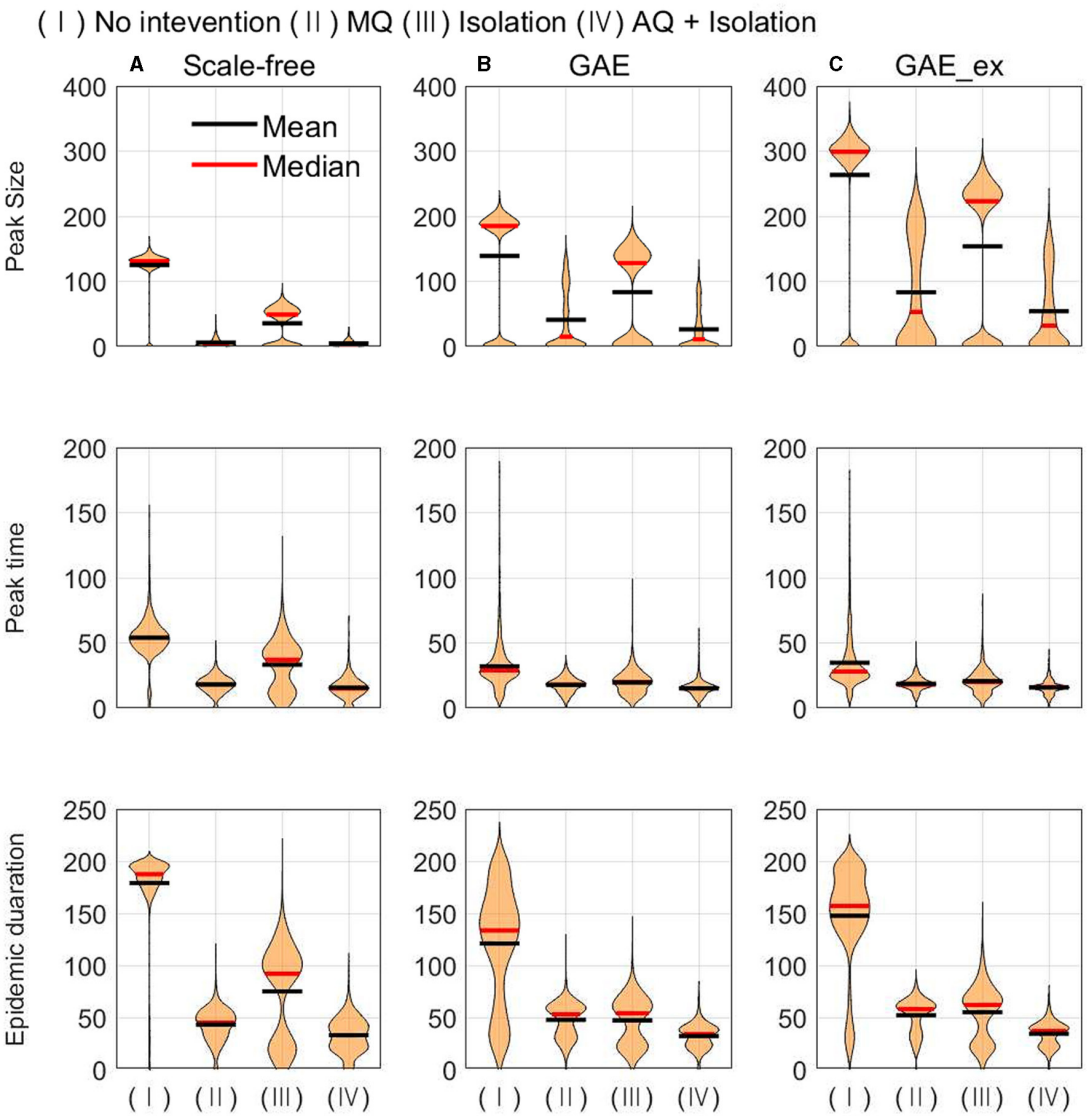
Peak size (top row), peak time (middle row), and epidemic duration (bottom row) under different intervention scenarios and on different networks (for each intervention, the result of 1,000 runs is shown). **(A)** SF, **(B)** GAE, and **(C)** GAE_ex.

In this subsection, the mean, median, and distributions of 1,000 simulation runs are summarized in terms of five epidemic outputs. The final size (cumulative incidence) and the total number of quarantined individuals for each network structure for different intervention strategies are presented in Figure 8. In addition, the peak size, peak time, and epidemic duration are summarized in Figure 9.

Figure 8 shows that the final size gets larger in the order of SF, GAE, and GAE_ex. Notably, the variances are smaller in SF than in GAE and GAE_ex. In addition, there are weak bi-modes in GAE and GAE_ex. For instance, for No intervention, SF shows mean and median of around 5,000, respectively, with a very small variance. Meanwhile, GAE shows median and mean around 6,000 and 4,000, respectively (most of the results are around 6,000 and some are around 100), and GAE_ex shows median and mean around 9,000 and 8,000, respectively (most of the results are around 9,000 and some are around 100). The impacts of the three intervention strategies are distinct on the final size distributions. Again, there is a very small variance in SF and weak bi-modes in GAE and GAE_ex, which can be explained by the distributions of index cases and clustering coefficients (Figure 3D).

Comparing the effectiveness of MQ and AQ + Isolation, both intervention strategies dramatically reduced the final size in SF (the first panel at the top row of Figure 8). However, the total quarantine size significantly differed; MQ quarantined around 25,000 individuals, whereas AQ + Isolation quarantined only 2,000 individuals (the first panel at the bottom row of Figure 8). Thus, AQ + Isolation can be a more effective strategy than MQ in SF. This is also the case in GAE and GAE_ex because similar patterns are observed. Although the means of cumulative quarantined individuals are similar in all three networks, the overall effectiveness of SF and GAE are quite different.

In Figure 9, the impacts of intervention strategies on different networks are compared in terms of the peak size, peak time, and epidemic duration. The results in the first row show that the peak size is the largest under No intervention and the second largest for Isolation in all networks. AQ + Isolation shows the smallest peak size in all networks. Peak size manifested a small variance in SF and weak bi-modes in GAE and GAE_ex, as the final size did in Figure 8; the mean and median are almost the same in SF, whereas they are quite different in GAE and GAE_ex. The results in the second row show that the mean and median of peak time distributions for all intervention strategies are very similar in GAE and GAE_ex. In addition, the mean, and median of SF are in the order of No intervention, Isolation, MQ, and AQ + Isolation, attributable to the shortest path (Figure 3B). The results in the third row suggest that the impacts of interventions are very different among networks. Under No intervention, outbreaks lasted about 180 days in SF with a very small variance. However, outbreaks lasted about 150 days in GAE and GAE_ex with very large variances. Notably, epidemic duration is longer for Isolation than for the other two intervention strategies in SF.

**Figure 10:**
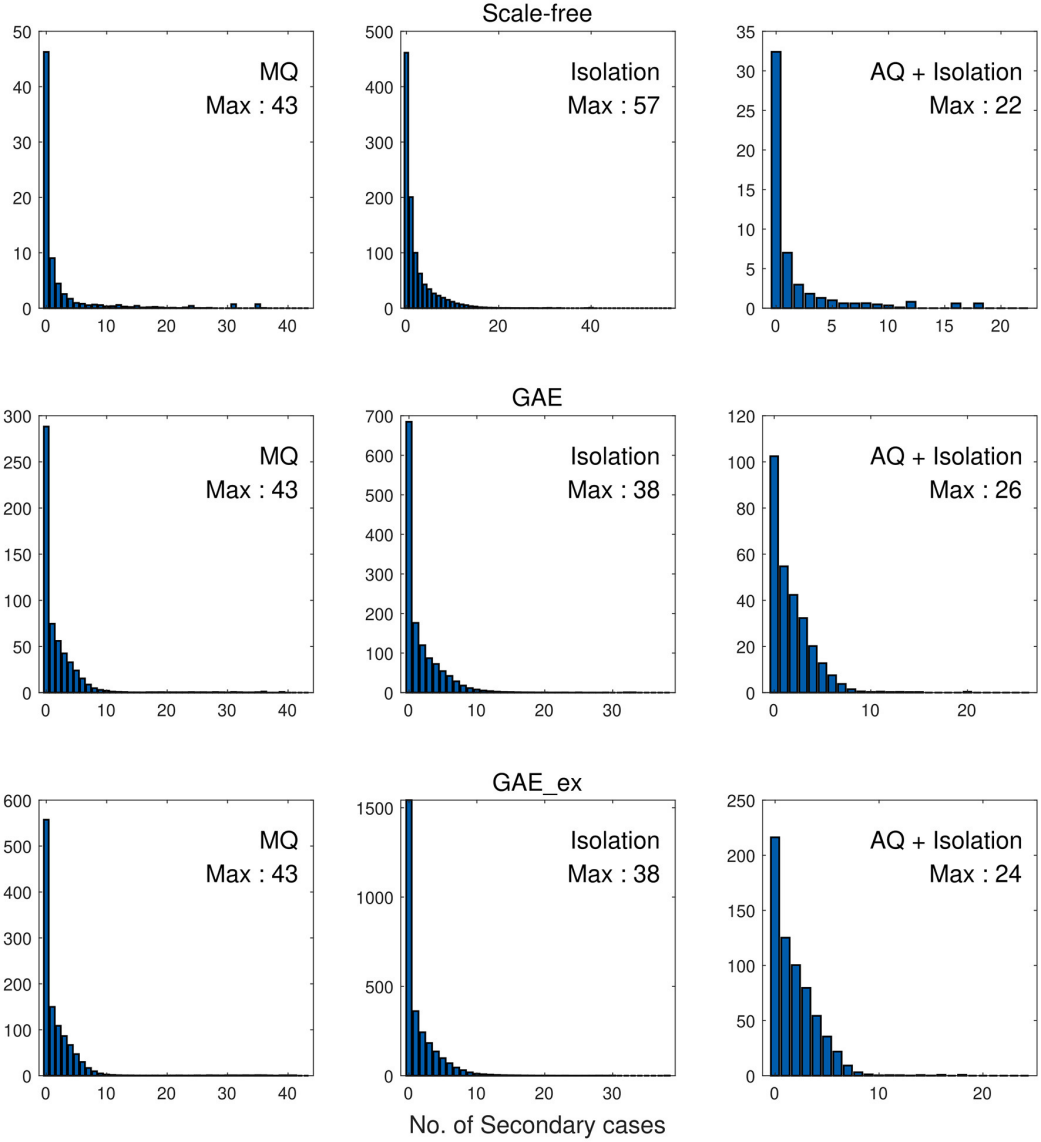
Distributions of secondary cases under different intervention scenarios and on different networks.

**Figure 11:**
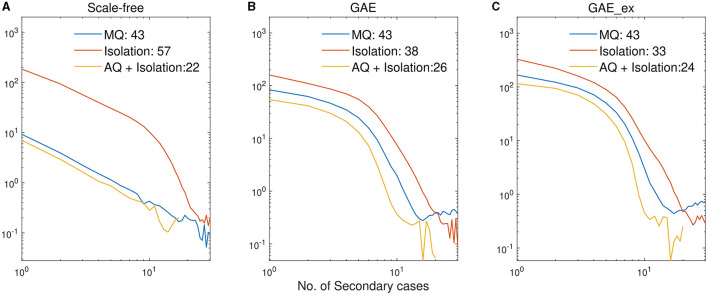
Log-log plots of distributions of secondary cases in Figure 10. **(A)** SF, **(B)** GAE, and **(C)** GAE_ex.

Finally, we investigate the impacts of the three intervention strategies on the distributions of secondary cases in each network structure. The mean distribution of 1,000 simulation runs is presented in Figure 10. An outbreak can be considered a super-spreading event when its distribution of secondary cases exhibits a large degree of heterogeneity. Each panel illustrates the type of intervention strategy and the maximum number of secondary cases by a single infected individual (denoted by Max). In fact, during the MERS-CoV outbreak in South Korea in 2015, a single patient infected 79 other individuals, referred to as a super-spreader (42). The results in Figure 10 suggest that Isolation is the least effective strategy to prevent super-spreading events on all networks. For instance, Max = 57 in SF, which is worse than Max = 38 in GAE and GAE_ex. AQ + Isolation showed the lowest level of heterogeneity in the secondary cases, with Max of 22, 26, and 24 in SF, GAE, and GAE_ex, respectively. Furthermore, Figure 11 provides a log–log representation of Figure 10, allowing for a comparison of the secondary cases for three interventions across various network configurations. This analysis verifies that Isolation alone (depicted by the red curves) consistently yields the highest values, signifying its limited effectiveness, while AQ + Isolation (indicated by the yellow curves) consistently exhibits the lowest values, highlighting its superior effectiveness across all network structures.

## 4 Discussions

We have employed an innovative graph autoencoder technique to recreate the contact network using real-world contact-tracing data. This marks the first utilization of such an approach for reconstructing networks based on contact-tracing data derived from the 2015 MERS-CoV outbreak in South Korea. We conducted a comparative analysis between the reconstructed networks and a scale-free network (SF) concerning the effectiveness of various intervention strategies. Furthermore, we explored the influence of network structure on epidemic outcomes, including peak size, final size, incidence, and cumulative incidence. The study's findings revealed that the severity of outbreaks followed the order of SF, GAE, and GAE_ex. Moreover, GAE and GAE_ex exhibited higher variances in both incidence and cumulative incidence compared to SF.

We also evaluated the impact of different intervention strategies, such as mass quarantine (MQ) and acquaintance quarantine (AQ) + isolation, on epidemic outputs in simulations on different networks. First, the results showed that MQ was found to be an equally effective strategy as AQ + isolation in the SF network. However, the study found that although the average shortest paths were similar in the three networks, the SF network was less influenced by hub nodes and had a wider distribution of shortest paths. We found that the effectiveness of MQ and AQ+Isolation in the SF network was excessively good, which is attributed to the low clustering coefficient of SF. The low clustering coefficient means that the SF network is less dense, which makes it more sensitive to interventions like MQ and AQ+Isolation.

Moreover, isolation was the least effective strategy in SF. This is due to the shortest path length and isolation of confirmed cases only which do not take pre-symptomatic cases into account. The Scale-free network is a type of network structure that is often used to model the spread of infectious diseases with a long tail distribution. This means that a small number of individuals (referred to as “super-spreaders”) are responsible for a large proportion of transmission. However, it has been found that this structure can lead to over estimations of the impact of interventions, as they may not be able to effectively target the super-spreaders.

It is important to note that the peak time of the outbreaks in the GAE and GAE_ex networks did not differ significantly when comparing the results of no intervention to those of the various intervention strategies. This is because the high clustering coefficients in GAE and GAE_ex lead to similar peak times. This is because nodes with low clustering coefficients do not typically experience outbreaks, while outbreaks are likely to occur in nodes with high clustering coefficients. Thus, outbreaks in GAE and GAE_ex spread uniformly throughout the network, which is different from the SF network, where the clustering coefficients and shortest path lengths are different. This trend is due to the unique characteristics of the GAE and GAE_ex networks, which are more similar to the contact networks found in hospital settings, specifically, emergency rooms in South Korea, and have higher clustering coefficients which implies higher density in most emergency rooms of hospitals in South Korea (55).

This study has several limitations worth noting. Firstly, it does not comprehensively explore the influence of index cases on the outbreak of the epidemic. In our simulations, index cases were randomly chosen from nodes with a sufficient number of connections. However, selecting super-spreaders identified in the contact-tracing data as index cases might produce different outcomes. Additionally, we refrained from conducting comparisons with other reconstruction methods. Analyzing our graph auto encoder approach alongside alternative link prediction models like Exponential Random Graph Model (ERGM) (56) and Bayesian statistical models (57) might have provided valuable insights into the effectiveness of our model. Furthermore, our study highlights a high clustering coefficient in the reconstructed network due to the concentrated distribution of emergency rooms in South Korea. This observation may not be applicable to other regions. Additionally, since our study utilized contact-tracing data from the 2015 MERS-CoV outbreak in South Korea, the generalizability of our findings is limited. Utilizing contact-tracing data from other outbreaks could lead to more universally applicable results. Moreover, future research could explore the impact of population mobility, as it is widely recognized that mobility plays a significant role in disease transmission (58). Incorporating mobility into simulation models could offer valuable insights.

## 5 Conclusions

We employed an innovative graph autoencoder technique to reconstruct the contact network using real-world contact-tracing data. This marks the first instance of utilizing such an approach to reconstruct networks based on contact-tracing data from the 2015 MERS-CoV outbreak in South Korea, which were subsequently employed in epidemic simulations. Our investigation focused on five key epidemic outcomes, conducting a comparative analysis of various network structures and intervention strategies. Our findings underscore the significant impact of network structures on epidemic outcomes, emphasizing the variable effectiveness of intervention strategies across different contexts. These findings carry significant implications for tailoring precise intervention measures in response to disease outbreaks.

Our results reveal substantial differences in the impacts of various network structures on epidemic outputs: outbreaks were more extensive in the scale-free network, a widely used theoretical model for epidemic simulation, compared to the reconstructed network generated by the link prediction method. Consequently, the effectiveness of intervention strategies can vary depending on the network structure: intervention measures on the reconstructed network were found to be less effective than those on the scale-free network. These results suggest a potential overestimation of intervention impact in scale-free networks, while our reconstructed network offers a more realistic assessment of intervention effectiveness. In this study, we opted for a scale-free network structure due to its suitability for meaningful comparisons compared to other models such as random networks. However, it is crucial to acknowledge the limitations associated with scale-free networks, as discussed earlier. Thus, to account for potential biases in our analysis, we emphasize the importance of recognizing the increased risk of overestimation when utilizing scale-free networks, as their structure may influence intervention outcomes. Therefore, the consideration of networks constructed through alternative methods, such as small-world networks or spatial networks, may introduce variability in the results (1, 14).

The utilization of networks reconstructed through link prediction methods proves to be a valuable asset for conducting epidemic simulations. In the specific context of the 2015 MERS-CoV outbreak in South Korea, this study leveraged this approach to reconstruct the contact-tracing network, aiming to evaluate the effectiveness of intervention strategies. We anticipate that this innovative methodology will inspire future endeavors aimed at enhancing simulation environments, providing valuable insights to guide the decisions of public health authorities. Moreover, it has the potential to stimulate further research to enhance the realism of simulation environments through data-driven network reconstruction methods.

## Data availability statement

The datasets presented in this article are not readily available because, this data was provided by the Korean Disease Control and Prevention Agency and is not publicly available. Therefore, this data should not be disclosed for purposes other than research. Requests to access the datasets should be directed to: https://www.kdca.go.kr/.

## Author contributions

EK: Conceptualization, Data curation, Formal analysis, Funding acquisition, Investigation, Methodology, Project administration, Resources, Software, Supervision, Validation, Visualization, Writing—original draft, Writing—review & editing. YK: Conceptualization, Data curation, Formal analysis, Funding acquisition, Investigation, Methodology, Project administration, Resources, Software, Supervision, Validation, Visualization, Writing—original draft, Writing—review & editing. HJ: Conceptualization, Data curation, Formal analysis, Funding acquisition, Investigation, Methodology, Project administration, Resources, Software, Supervision, Validation, Visualization, Writing—original draft, Writing—review & editing. YL: Conceptualization, Data curation, Formal analysis, Funding acquisition, Investigation, Methodology, Project administration, Resources, Software, Supervision, Validation, Visualization, Writing—original draft, Writing—review & editing. HL: Writing—original draft, Writing—review & editing. SL: Conceptualization, Data curation, Formal analysis, Funding acquisition, Investigation, Methodology, Project administration, Resources, Software, Supervision, Validation, Visualization, Writing—original draft, Writing—review & editing.
